# Suppression of Early TNF-Alpha Increase by a Single Evolocumab Dose in Patients with Acute Myocardial Infarction Undergoing Percutaneous Coronary Intervention

**DOI:** 10.3390/jcm15134873

**Published:** 2026-06-23

**Authors:** Giuseppe Patti, Manuel Bosco, Alessandra Marengo, Luca Cumitini, Leonardo Grisafi, Domenico D’Amario, Martina Solli, Marco Mennuni

**Affiliations:** 1 Department of Translational Medicine, University of Eastern Piedmont, Via Solaroli 17, 28100 Novara, Italy; 2 Division of Cardiology, Maggiore della Carità Hospital, 28100 Novara, Italylucacumitini@gmail.com (L.C.);

**Keywords:** acute myocardial infarction, percutaneous coronary intervention, evolocumab, PCSK9 inhibition, inflammation, TNF-alpha, lipid-lowering therapy

## Abstract

**Background:** Early initiation of proprotein convertase subtilisin/kexin type 9 inhibitors (PCSK9-i) in patients with acute myocardial infarction (MI) may anticipate and maximize lipid-lowering benefit. Whether PCSK9 inhibition also exerts early anti-inflammatory effects in this setting remains unclear. This study aimed to evaluate the effects of early PCSK9-i administration on inflammatory markers and lipid parameters in patients with acute MI undergoing percutaneous coronary intervention (PCI). **Methods:** In this randomized, prospective, single-center, open-label trial, patients with acute MI undergoing PCI were randomly assigned to receive a single upstream 140 mg subcutaneous dose of evolocumab immediately before PCI, on top of oral lipid-lowering therapy (LLT) (n = 30), or oral LLT alone (control group; n = 30). Tumor necrosis factor-alpha (TNF-α), low-density lipoprotein cholesterol (LDL-C), apolipoprotein B (apoB), and lipoprotein(a) [Lp(a)] levels were measured at baseline and during the early post-intervention phase. **Results:** Baseline TNF-α values and lipid parameters were similar between the two groups. At 72 h after PCI, TNF-α levels were significantly lower in the evolocumab arm compared with controls (0.01 vs. 0.25 pg/mL; *p* = 0.025). Evolocumab was also associated with a greater relative reduction in LDL-C levels from baseline (−48% vs. −18%; *p* < 0.001) and apoB levels (−34% vs. −11%; *p* < 0.001). The proportion of patients achieving the LDL-C goal of <55 mg/dL at 72 h was higher in the evolocumab group than in controls (50% vs. 10%; *p* < 0.001). Lp(a) levels at 72 h were also lower with evolocumab (12 [10–33] vs. 28 [13.1–70] mg/dL; *p* = 0.032). **Conclusions:** In patients with acute MI undergoing PCI, upstream administration of a single evolocumab dose was associated with suppression of the early post-intervention increase in TNF-α levels, together with rapid reductions in LDL-C, apoB, and Lp(a). These findings suggest a potential modulation of the early inflammatory response by PCSK9 inhibition in addition to its lipid-lowering effects. Larger studies are needed to confirm these observations and to determine their clinical relevance.

## 1. Introduction

Low-density lipoprotein cholesterol (LDL-C) lowering represents a cornerstone of secondary prevention in patients with acute coronary syndrome (ACS), with the clinical benefit being proportional to the degree of LDL-C reduction [1]. Proprotein convertase subtilisin/kexin type 9 inhibitors (PCSK9-i), including evolocumab and alirocumab, potently reduce circulating LDL-C levels by preventing LDL receptor degradation, offering a key lipid-lowering option alongside oral treatments [2]. The use of PCSK9-i reduced the incidence of major adverse cardiovascular events (MACE) in patients with recent ACS or atherosclerotic cardiovascular disease (ASCVD) and elevated LDL-C values despite oral lipid-lowering therapy (LLT) [3,4], without safety concerns, even when very low LDL-C levels were reached [5]. The 2019 European Society of Cardiology (ESC)/European Atherosclerosis Society (EAS) guidelines recommended in patients with ACS an LDL-C target <55 mg/dL, supporting a stepwise therapeutic approach to achieve this goal [6]. However, real-world data showed that such an approach is associated with low percentages of patients at target for LDL-C during follow-up [7,8]. Thus, recent European consensus documents and real-world data suggested a “strike early and strong” strategy, which advocates in patients with ACS for an early initiation of intensive, combination LLTs. Including a prompt use of PCSK9-i, with the aim of achieving the LDL-C goal more quickly and in a large number of patients, anticipates the clinical benefit [9,10,11].

This newer strategy is based on investigations highlighting the effectiveness of an early PCSK9-i administration in patients with ACS in terms of early and strong LDL-C reduction and consequent stabilizing effects on the coronary plaques [12,13,14]. A reduction in inflammatory cells within coronary plaques was demonstrated with prolonged PCSK9-i use, and this was considered mainly as a consequence of LDL-C reduction [13]. However, PCSK9 might directly promote changes in atherosclerotic plaque composition by triggering an inflammatory response where cytokines, chemokines, and adhesion molecules are involved [15]. Recent studies demonstrated in the animal model that PCSK9-i exhibit anti-inflammatory effects—particularly, reduction in macrophage accumulation and suppression of pro-inflammatory cytokine production [16,17]. However, to date, no investigation has addressed the presence of early, direct anti-inflammatory effects after PCSK9-i use in the human model. Thus, we designed a randomized trial to evaluate the impact of a single evolocumab dose, given immediately before percutaneous coronary intervention (PCI), on early inflammatory markers and lipid levels in patients admitted for ST-segment elevation myocardial infarction (STEMI) or non-STEMI (NSTEMI). Data on this topic are relevant, as they may provide insights into the benefit of upstream administration of PCSK9-i in these high-risk populations in terms of inflammatory burst attenuation and potential outcome improvement through pleiotropic effects.

## 2. Methods

### 2.1. Study Design

This randomized, prospective, single-center, open-label trial was conducted at the Division of Cardiology, Maggiore della Carità Hospital in Novara, Italy. The study protocol was approved by the local Institutional Ethics Committee (approval numbers CE272/2023 and E00026/2024) and conducted in accordance with Declaration of Helsinki and Good Clinical Practice guidelines. Written informed consent was obtained from all participants. Patients were included from September 2023 to September 2024.

### 2.2. Study Population

Inclusion criteria were: patients ≥18 years presenting with ST-segment elevation myocardial infarction (STEMI), defined as symptoms of myocardial infarction lasting ≥30 min within the past 24 h, with new persistent ST-segment elevation ≥1 mm in ≥2 continuous electrocardiographic leads and treated with primary PCI, or with non−ST-segment elevation myocardial infarction (NSTEMI) and symptom onset ≤72 h, receiving early (<24 h) coronary angiography and PCI. Exclusion criteria were: (a) hemodynamic instability (Killip Class III/IV or sustained symptomatic hypotension with systolic blood pressure <80 mmHg); (b) fibrinolysis treatment; (c) indication to coronary artery bypass grafting after coronary angiography; (d) known creatinine clearance <30 mL/min; (e) severe liver disease; (f) active malignancy or estimated life expectancy ≤12 months; (g) prior or ongoing PCSK9-i therapy.

### 2.3. Randomization, Treatment Allocation and Blinding

If PCI was considered to be indicated and the patient met the enrollment criteria, after coronary angiography, they were assigned in a 1:1 ratio to receive either a single 140 mg subcutaneous evolocumab dose given immediately prior to PCI (active treatment arm) or no evolocumab administration (control arm). Treatment assignment was done using pre-specified randomization blocks. Randomization was performed independently of baseline LDL-C levels and prior or current LLT. Due to the nature of the intervention, the study was conducted in an open-label fashion. Patients and treating clinicians were aware of the treatment allocation. However, laboratory personnel performing biochemical analyses, as well as investigators performing statistical analyses, were blinded to the treatment assignment.

### 2.4. Study Measurements and Follow-Up

Immediately after percutaneous revascularization, blood samples were collected to measure levels of total cholesterol, LDL-C, high-density lipoprotein cholesterol (HDL-C), triglycerides (TG), apolipoprotein B (apoB), Lipoprotein(a) [Lp(a)], Tumor Necrosis Factor-alpha (TNF-α), E-selectin and high-sensitivity C-reactive protein (hs-CRP). The measurement of LDL-C, TNF-α, E-selectin and hs-CRP values was repeated at 24 and 72 h, whereas levels of apoB and Lp(a) were re-assessed at 72 h after PCI. The central laboratory remained blinded to the treatment allocation. After collecting blood samples, they were centrifuged at 3600 rpm for 10 min, and plasma and serum were separated into aliquots and stored at −80 °C. Serum TNF-α levels were measured by a commercially available Human TNF-α ELISA kit (Diaclone, Besançon, France, Cat. No. 950.090.096, batch 1100-135) on a SKYLAB 752 platform (DASIT), according to the manufacturer’s instructions. The assay had a manufacturer-reported minimum detectable dose of 8 pg/mL and a calibration range of 25–800 pg/mL, while no specific lower limit of quantification (LLOQ) was provided. TNF-α concentrations were recorded as generated by the original SKYLAB 752 software output, without any post-analytical imputation or replacement of low values. E-selectin was measured using the PANTEC enzymatic immunoassay (ELISA), with analysis performed on a SKYLAB 752 instrument (DASIT, Cornaredo, Milan, Italy). Lipid parameters and hs-CRP were analyzed in plasma by the Cobas C702 Chemistry Analyzer (Roche Diagnostics, Risch, Switzerland).

In both arms, oral LLT was amended the same day of the procedure, with drug type and dose being established according to baseline LDL-C levels and previous chronic LLT. Participants in the active treatment arm subsequently received evolocumab 140 mg every two weeks. Patients in the control group who were considered unable to achieve the LDL target of <55 mg/dL with oral LLT, received a PCSK9-i at discharge, after at least 72 h from PCI, which was then given every two weeks and were included in the 30-day analyses according to the intention-to-treat principle. As evolocumab was initiated after the completion of the 72 h assessments, these patients did not contribute to the primary endpoint analysis or to the evaluation of early post-PCI lipid and inflammatory outcome measures. All patients were treated with the other medical therapies for ACS in accordance with current standard of care. A follow-up visit was planned at 30 days post-discharge, when clinical data were collected and a blood sample for LDL-C measurement was obtained.

### 2.5. Outcomes

Primary endpoint was the comparison of TNF-α levels between the two arms (evolocumab vs. controls) at 72 h post-PCI.

Secondary endpoints were:

- Comparisons of LDL-C and apoB levels between the two arms (evolocumab vs. controls) at 72 h post-PCI;
- Reductions in LDL-C levels from baseline at 24 and 72 h;
- Percentage of patients achieving the LDL-C goal (<55 mg/dL) at 72 h;
- Comparisons of Lp(a), hs-CRP and E-selectin levels between the two arms at 72 h;
- Clinical outcome and percentage of patients achieving the LDL-C goal (<55 mg/dL) at 30 days.

Additional analyses were performed to assess the impact of early evolocumab use on TNF-α levels according to clinical presentation (NSTEMI vs. STEMI) and on LDL-C decrease at 72 h according to prior statin therapy or not.

### 2.6. Statistical Analysis and Sample Size Calculation

Categorical variables are presented as absolute numbers (percentages). Continuous variables are expressed as mean ± standard deviation (SD) or median [interquartile range (IQR)], based on their distribution, which was assessed using the Shapiro–Wilk test. Comparisons between the two arms were performed by Student’s *t*-test for normally distributed continuous variables and the Mann–Whitney U test for non-normally distributed continuous variables. For categorical variables, the Fisher exact test was used if the expected frequency was <5, otherwise the chi-square test (χ^2^) was utilized. For repeated measurements at different time points within the same arm, changes were assessed by the Friedman test for variables measured at ≥3 time points; for variables assessed at 2 time points only, within-group comparisons were performed by the Wilcoxon signed-rank test. Absolute reductions in laboratory parameters were calculated by subtracting the 24 h or 72 h values from baseline levels. Relative reductions were determined by dividing the absolute reductions by baseline values. Given the exploratory and pilot nature of this study, no prior randomized data were available to support a formal sample size calculation based on early TNF-α levels changes as primary endpoint in the specific setting of patients with ACS undergoing PCI. Therefore, the sample size was based on TNF-α values reported in other STEMI populations. In particular, using the median and interquartile range reported by Gonzálvez et al. for TNF-α in STEMI patients [18], the standard deviation was approximated as IQR/1.35: (2.92 − 1.39)/1.35 = 1.13 pg/mL. As the distribution showed a positive skewness (upper semi-IQR: 0.90 pg/mL > lower semi-IQR: 0.63 pg/mL), IQR/1.35 might underestimate the true SD. To account for this uncertainty, the power target was conservatively increased from 80% to 90%. Assuming a hypothesized 50% relative reduction in TNF-α with PCSK9 inhibition (estimated by analogy with the 13% to 23% reduction in cell adhesion molecules observed with atorvastatin pre-treatment in the ARMYDA-CAMs substudy [19], and assuming a greater pleiotropic effect for PCSK9 inhibitors), corresponding to an absolute difference of 1.01 pg/mL, with a two-sided α = 0.05 and 90% power, the estimated sample size was 27 patients per arm using a z-test approximation and 28 patients per arm using a two-sample *t*-test. Thus, the enrollment of 30 patients per arm was planned, also accounting for potential dropouts. All *p*-values were two-sided, with a significance threshold <0.05. Statistical analyses were performed using STATA version 18.0 (StataCorp, College Station, TX, USA).

## 3. Results

As indicated in the graphical diagram (Supplementary Figure S1), a total of 64 participants were enrolled and randomized to receive evolocumab on top of oral LLT (n = 33) or LLT alone (n = 31, control arm). Following PCI, a total of 4 patients were excluded early (<24 h)—3 in the active treatment group and 1 in the control group—for: acute and severe hemodynamic instability (n = 1); post-procedural death (n = 1); consent withdrawal (n = 1); technical issues causing unavailability of the laboratory sample analysis (n = 1). Thus, a total of 60 patients represented the final study population (30 in each arm) (Supplementary Figure S1). Baseline characteristics, including demographic features, risk factors, clinical status, comorbidities, concomitant treatments, laboratory parameters and background LLT, were similar in the two arms (Table 1). In particular, mean age was 67.3 ± 11.1 years in the evolocumab group vs. 67.1 ± 9.5 years in controls, prevalence of female gender was 16.7% in both groups and percentage of STEMI presentation was 26.7% vs. 40%. The frequency of prior statin therapy was 40% vs. 26.7% (*p* = 0.27). Baseline LDL-C levels were 106 [76–130] mg/dL in the evolocumab arm vs. 95 [79–112] mg/dL in the control arm (*p* = 0.37), with median apoB levels being 81 [62–107] mg/dL vs. 78 [68–96] mg/dL, respectively (*p* = 0.96) (Table 2). Other lipid parameters were also similar (Table 1). TNF-α levels at baseline were comparable (0.01 [0.01–0.01] pg/mL in both arms; *p* = 0.84) (Table 2). After PCI, 93.3% of patients in the evolocumab group and 100% in the control group received high-intensity statin therapy. All patients in the evolocumab arm continued the drug after discharge, whereas 2 patients in the control group were given evolocumab at discharge in addition to statin and ezetimibe, due to the expected inability to reach the goal with oral LLT alone.

### 3.1. Outcome Measures

**Figure 1:**
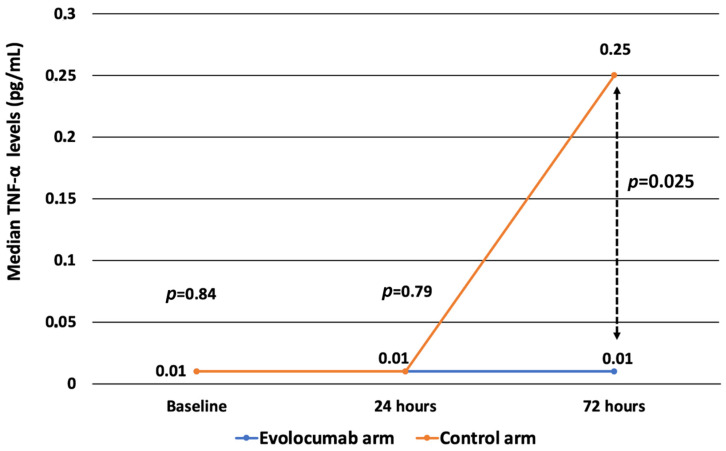
Median TNF-α levels at baseline, 24 h and 72 h in the two arms. TNF-α = Tumor Necrosis Factor-alpha.

At 24 h post-PCI, TNF-α levels were unchanged and similar in the two arms (Table 2; Figure 1). At 72 h, TNF-α values remained suppressed in the evolocumab group (0.01 [0.01–0.01] pg/mL), whereas they were raised significantly in controls (0.25 [0.01–4.56] pg/mL; *p* = 0.025 between the two arms). These data were confirmed by longitudinal measurements within each arm. In fact, within-group longitudinal analyses across 3 time points (by the Friedman test) showed a significant over time increase in TNF-α levels after PCI in the control arm (χ^2^ = 13.56, *p* = 0.001), whereas no significant change was observed in the evolocumab arm (χ^2^ = 1.22, *p* = 0.544) (Supplementary Table S1). Within-group longitudinal analyses across 2 time points (by the Wilcoxon signed-rank test) confirmed stable over time TNF-α levels in the evolocumab group (all *p* > 0.1), whereas in the control group a borderline significant increase was observed at 72 h vs. baseline (*p* = 0.053) (Supplementary Table S2).

**Figure 2:**
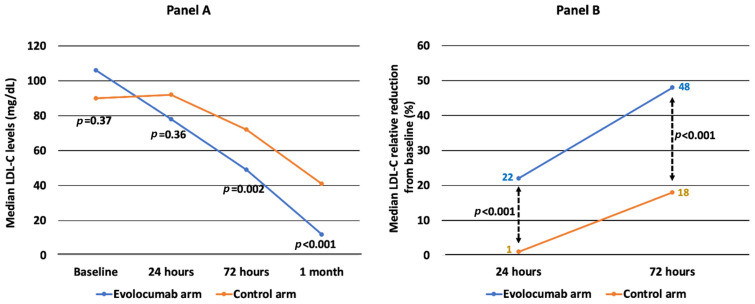
Panel (**A**): Median LDL-C levels at baseline, 24 h, 72 h and 1 month in the two arms. Panel (**B**): Median relative reduction from baseline of LDL-C levels at 24 h and 72 h in the two arms. LDL-C = Low-Density Lipoprotein Cholesterol.

**Figure 3:**
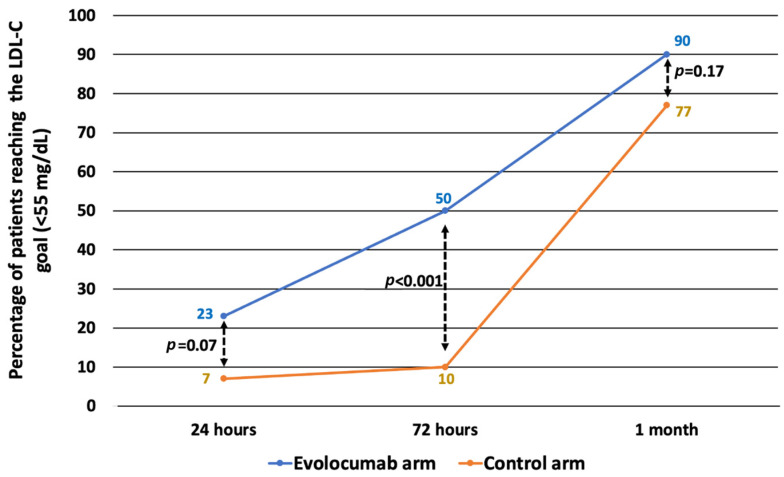
Percentage of patients reaching the LDL-C goal (<55 mg/dL) at 24 h, 72 h and 1 month in the two arms. LDL-C = Low-Density Lipoprotein Cholesterol.

**Figure 4:**
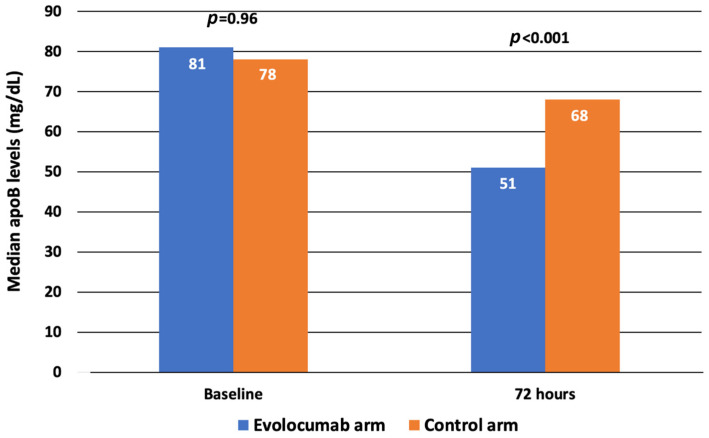
Median ApoB levels at baseline and 72 h in the two arms. ApoB = Apolipoprotein B.

The relative reduction in LDL-C from baseline in the evolocumab vs. control arm was already significant at 24 h post-PCI (−22% vs. −1%, *p* < 0.001) (Table 2; Figure 2) and was even greater at 72 h (−48% vs. −18%, *p* < 0.001), when with evolocumab use a higher proportion of patients achieved the LDL-C goal (50% vs. 10% in controls, *p* < 0.001) (Figure 3) and absolute LDL-C levels were lower (49 [35–70] vs. 72 [60–92] mg/dL, *p* = 0.002) (Table 2; Figure 2). In the evolocumab group, apoB levels were reduced at 72 h (51 [35–70] vs. 68 [60–83] mg/dL in controls, *p* < 0.001), when the relative reduction compared to baseline in the former was higher (−34% vs. −11%, *p* < 0.001) (Table 2; Figure 4).

**Figure 5:**
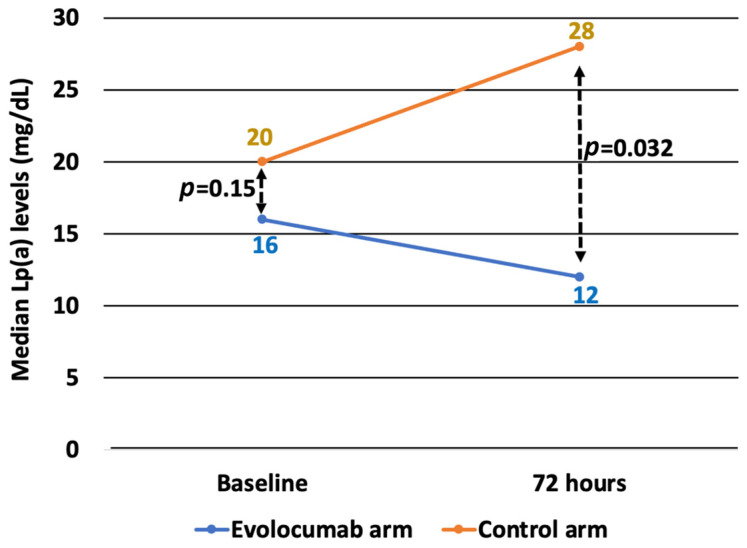
Median Lp(a) levels at baseline and 72 h in the two arms. Lp(a) = Lipoprotein(a).

Baseline Lp(a) levels were similar in the two arms (16 [10–30] mg/dL with evolocumab vs. 20 [11.5–57] mg/dL in controls, *p* = 0.15) (Table 3; Figure 5). At 72 h, Lp(a) levels decreased in the active treatment group and increased in the control group (12 [10–33] mg/dL and 28 [13.1–70] mg/dL, respectively; *p* = 0.032 between the two arms).

Hs-CRP levels at 24 h and 72 h were not attenuated by evolocumab and were similar between the two groups at any post-PCI determination (Table 3). Within each group, hs-CRP levels post-PCI increased significantly vs. baseline across 3 time points analyses (Friedman *p* < 0.001 for both arms) (Supplementary Table S1). Wilcoxon tests confirmed across 2 time points significant within-group increases at 24 and 72 h vs. baseline in both arms (*p* ≤ 0.033) (Supplementary Table S2). Post-procedural E-selectin levels remained essentially unchanged compared to baseline and were similar in the two groups (Table 3).

At 1 month, patients in the evolocumab arm showed lower absolute LDL-C levels (12 [8–18] vs. 41 [33–50] mg/dL, *p* < 0.001), but did not have a significantly higher achievement of the LDL-C goal (90% vs. 77% in controls, *p* = 0.17) and (Table 4). Two patients in the control group initiated evolocumab at discharge because the achievement of the LDL-C target with oral lipid-lowering therapy alone was considered unlikely. In a sensitivity analysis, by excluding these two patients, LDL-C levels at 1 month remained significantly lower in the evolocumab group (12 [8–18] vs. 41 [33–52] mg/dL in the control group, *p* < 0.001). At 1 month, no patient in the two arms had cardiovascular events. One patient in the evolocumab group died one week after PCI due to sepsis.

### 3.2. Subgroup Analyses

The reduction in TNF-α at 72 h with evolocumab was regardless of clinical presentation (NSTEMI or STEMI) (Supplementary Table S3). At 72 h, the relative reduction in LDL-C in the evolocumab arm vs. controls was significant both in statin-naïve patients (−44% vs. −21%, *p* < 0.001) and in those on chronic statin therapy (−54% vs. −7%, *p* = 0.010).

## 4. Discussion

This randomized study shows a suppression of post-procedural TNF-α levels increase at 72 h by upstream evolocumab initiation in patients with acute MI undergoing PCI. A key novel aspect of the present study is the characterization of the early inflammatory response following upstream evolocumab administration through serial TNF-α levels assessment after percutaneous coronary revascularization for acute MI. In particular, after a single evolocumab dose, TNF-α levels remained stably suppressed over the first 3 days, whereas in the control group there was a post-PCI inflammatory burst, characterized by a marked elevation of TNF-α levels. These data suggest the potential benefit of an early evolocumab administration to attenuate the inflammatory response in patients with acute MI receiving percutaneous coronary revascularization. Moreover, evolocumab use was associated with significant reduction in LDL-C, apoB and Lp(a) levels at 72 h, when a higher rate of LDL-C goal achievement was obtained.

Inflammation plays a key role in the development and destabilization of atherosclerotic plaques, where PCSK9 may amplify pro-inflammatory processes [20]. Experimental studies indicated that, beyond their lipid-lowering effects, PCSK9-i administration reduces inflammation by limiting cytokine production, monocyte adhesion to endothelial cells and intra-plaque expression of TNF-α [21,22]. To the best of our knowledge, the present study is the first randomized trial to suggest an immediate direct anti-inflammatory action by PCSK9 inhibition in patients with acute MI undergoing PCI, as expressed by early suppression of TNF-α levels elevation. To date, available data are confined to a small retrospective cohort by Shimizu et al. [23], where TNF-α was assessed 15 days after admission, and to a pilot randomized study by Trankle et al. [24] (10 patients per arm) demonstrating no TNF-α reduction at 72 h after alirocumab administration.

During acute MI, the inflammatory response aimed to remove damaged cells. However, excessive inflammation can lead to additional harm [25]. TNF-α is a cytokine produced by cardiac myocytes and macrophages, and acts through both autocrine and paracrine mechanisms to perpetuate inflammation. In the myocardial tissue, TNF-α induces apoptosis of myocytes and endothelial cells, promotes oxidative stress and recruits neutrophils and macrophages [26]. Notably, elevated TNF-α levels after MI were associated with increased risk of cardiovascular events, as the post-event inflammation can lead to secondary damage causing an adverse myocardial remodeling [27]. Moreover, in patients undergoing PCI, especially for ACS, a post-procedural inflammatory response has been demonstrated, with subsequent impairment of clinical outcome being proportional to the intensity of such response [28,29,30]. Thus, targeting the early inflammatory burst post-MI by mitigating its contribution to myocardial damage might improve prognosis. Recently, this has been investigated in specific randomized studies with anakinra, an Interleukin-1 (IL-1) receptor antagonist [31], and with IL-6 receptor blockade by tocilizumab [32].

The early suppression of TNF-α observed in our investigation indicates that, when given in the acute phase of MI, a single evolocumab dose might have pleiotropic effects in terms of inflammation reduction. This suggests a possible therapeutic benefit related to mechanisms independent of lipid-lowering actions, able to impact on inflammatory processes driving post-infarction myocardial injury. While statins are known for their short-term pleiotropic effects providing a clinically relevant myocardial protection in patients with ACS undergoing PCI [33], lipid-lowering independent actions by PCSK9 inhibition in this setting were not previously described. Previous randomized studies indicated that pre-treatment with high-dose statin in patients undergoing PCI attenuates the procedural release of adhesion molecules, including E-selectin [34]. Indeed, we observed that post-procedural attenuation of the inflammatory burst by evolocumab load appears mainly related to decrease TNF-α release, without change in E-selectin levels. Importantly, in our study post-procedural hs-CRP levels were not acutely modified by upstream evolocumab administration. This expands previous findings, showing that clinical benefit in randomized trials and anti-inflammatory effects in experimental studies by PCSK9-i use were not associated with attenuation of hs-CRP levels [19,35]. Indeed, it has been demonstrated that protective, anti-inflammatory mechanisms are activated in the first days post-acute MI, with the aim to remove debris and dead cells, and promote healing of the damaged myocardium [25]. Such protective processes are mediated by production of IL-2 and IL-10, promoting the conversion of M1 (pro-inflammatory) slanted macrophages towards alternative M2 (anti-inflammatory) macrophages, with consequent reduction in pro-inflammatory mediators release, such as TNF-α, IL-8 and monocyte chemoattractant protein-1 [25]. Notably, such healing mechanisms do not have an impact on levels of CRP, that in patients with acute MI remains persistently elevated, and whose production is stimulated by IL-1 and IL-6.

With regard to lipid-lowering effects, the upstream evolocumab use provided a significant 22% LDL-C relative reduction already at 24 h post-intervention; such decrease was greater at 72 h (−48%), when one half of patients in the evolocumab arm achieved the recommended LDL-C goal. This pattern of LDL-C lowering in the evolocumab group was paralleled by similar attenuation of apoB levels. These features highlight the rapid and potent lipid-lowering effects of PCSK9-i also in the clinical setting of ACS [36] and align with recent evidence from the EVACS I trial, using evolocumab in patients with NSTEMI, and from the EPIC-STEMI trial, using alirocumab in those with STEMI [37,38]. In particular, our findings extend the early and potent lipid-lowering action by evolocumab across the entire spectrum of ACS (e.g., also in the STEMI patients, who were excluded in EVACS-I). These data provide a preliminary biological plausibility for the contemporary ‘strike early and strong’ strategy in patients with ACS, suggesting that an early PCSK9-i initiation can offer benefits beyond rapid lipid lowering through a potential modulation of the early inflammatory response [11].

Importantly, we found that Lp(a) levels at 72 h were increased from baseline in the control arm and were slightly reduced by evolocumab. Lp(a) is a low-density lipoprotein-like particle containing apoB linked to apolipoprotein(a) and contributes to atherosclerotic cardiovascular disease through pro-atherogenic, pro-inflammatory, and pro-thrombotic mechanisms [39]. Previous data demonstrated that a chronic PCSK9-i use lowers Lp(a) levels and part of the clinical benefit of this PCSK9 inhibition may be attributed to Lp(a) reduction, independently of LDL-C reduction [40]. Our results indicate that the Lp(a) decrease by evolocumab is also confirmed in ACS patients and occurs very early. Of note, we observed an increase in Lp(a) values at 72 h post-MI in the control arm; this further supports that Lp(a) can also act as an acute-phase protein [41].

The present study should be considered in light of its limitation. First, the open-label design may introduce a potential bias; however, all laboratory analyses were performed in a blinded fashion, reducing the risk of measurement bias for the biochemical outcome measures. Second, the relatively small sample size and the presence of potential confounders (for example the small difference in statin initiation rates between the two arms) limit the statistical power; moreover, our findings may be influenced by a high variability of inflammatory markers in the acute phase setting. Thus, the results of the present investigation should be considered exploratory and the observed associations do not prove causation. However, our data have the strength of a randomized design and a blind assignment of the laboratory outcome measures. Third, the sample size precludes the ability to perform robust subgroup analyses, which must be considered only exploratory and hypothesis-generating. Fourth, circulating TNF-α levels were extremely low, and a substantial proportion (maximum 80% at 72 h in the evolocumab arm and 73% at baseline in the control arm) of samples yielded values corresponding to the lowest value reported by the analytical system (0.01 pg/mL), below the assay detection limit. Accordingly, absolute TNF-α concentrations should be interpreted with caution. Although all values were obtained directly from the original ELISA Immunology kits output and no post hoc imputation was performed, the limited sensitivity of the assay may have affected the precision of TNF-α quantification. In addition, the clustering of values near the lower detection limit may have introduced analytical variability, and the observed between-group differences should therefore be considered exploratory. Fifth, the follow-up duration for TNF-α data was very short and further investigations are warranted to evaluate longer-term TNF-α variations. Finally, the effects of evolocumab on inflammatory parameters refer to patients with acute MI undergoing PCI. Thus, we are not able to describe such effects in patients with acute MI not receiving PCI (exploring if evolocumab reduces the inflammation strictly related to acute MI) or in patients treated with PCI not for acute MI (exploring if evolocumab reduces post-PCI inflammation regardless of clinical presentation of coronary artery disease).

## 5. Conclusions

Our findings suggest, for the first time, a possible in vivo dual mechanism of action of PCSK9-i, when given in patients with acute MI, such as anti-inflammatory actions beyond lipid-lowering effects. However, other investigations are needed to investigate the precise pathways involved. Moreover, given the exploratory nature of this study, the small sample size and the short follow-up duration, our results should be considered hypothesis-generating and require confirmation in larger, adequately powered, randomized trials to determine whether these early biological effects translate into meaningful clinical benefit.

## Figures and Tables

**Table 1:** Main characteristics in the two arms.

	Evolocumab Arm (n = 30)	Control Arm (n = 30)	*p* Value
Demographic characteristics			
Age (years)	67.3 ± 11.1	67.1 ± 9.5	0.95
Male gender	25 (83.3)	25 (83.3)	1.00
Cardiovascular risk factors and comorbidities			
Arterial hypertension	23 (76.7)	20 (66.7)	0.39
Diabetes mellitus	8 (26.7)	5 (16.7)	0.53
Dyslipidemia	22 (73.3)	20 (66.7)	0.57
PAD	4 (13.3)	5 (16.7)	1.00
COPD	2 (6.7)	1 (3.3)	1.00
Previous PCI	6 (20.0)	4 (13.3)	0.73
Previous CABG	2 (6.7)	1 (3.3)	1.00
Clinical presentation			
STEMI	8 (26.7)	12 (40.0)	0.27
NSTEMI	22 (73.3)	18 (60.0)	0.27
Angiographic/echocardiography data			
Multivessel disease	14 (47)	16 (53)	0.61
LVEF (%)	50.2 ± 6.7	49.5 ± 7.3	0.69
Laboratory data			
Hemoglobin (g/dL)	13.9 ± 2.0	14.0 ± 1.2	0.99
eGFR (mL/min/1.73 m^2^)	73.0 ± 24.2	74.5 ± 24.3	0.81
Total cholesterol (mg/dL)	157.9 ± 42.1	153.1 ± 43.2	0.67
HDL-C (mg/dL)	41.2 ± 10.7	39.4 ± 11.0	0.53
Triglycerides (mg/dL)	81.7 ± 35.1	85.1 ± 59.4	0.79
Antiplatelet therapy post-PCI			
Aspirin	30 (100)	30 (100)	
Ticagrelor	19 (63)	21 (70)	0.58
Clopidogrel	10 (33)	8 (27)	0.57
Lipid-lowering therapy before admission			
Statin	12 (40.0)	8 (26.7)	0.27
Ezetimibe	6 (20.0)	6 (20.0)	1.00
Lipid-lowering therapy at discharge			
Potent statin	28 (93.3)	30 (100)	0.42
Ezetimibe	25 (83.3)	25 (83.3)	0.76
PCSK9i	30 (100)	2 (6.7)	**<0.001**

Data are expressed as mean ± standard deviation for continuous variables (normal distribution) or as absolute numbers (percentages) for categorical variables. CABG = Coronary Artery Bypass Grafting; COPD = Chronic Obstructive Pulmonary Disease; eGFR = estimated Glomerular Filtration Rate; HDL-C = High-Density Lipoprotein Cholesterol; LVEF = Left Ventricular Ejection Fraction; NSTEMI = Non-ST-segment Elevation Myocardial Infarction; PAD = Peripheral Arterial Disease; PCI = Percutaneous Coronary Intervention; PCSK9i = proprotein convertase subtilisin/kexin type 9 inhibitors; STEMI = ST-segment Elevation Myocardial Infarction. Statistically significant *p* values (<0.05) appear in bold.

**Table 2:** Primary endpoints: TNF-α, LDL-C and apoB levels in the two arms.

	Evolocumab Arm (n = 30)	Control Arm (n = 30)	*p* Value
TNF-α levels			
Baseline (pg/mL)	0.01 [0.01–0.01]	0.01 [0.01–0.01]	0.84
24 h (pg/mL)	0.01 [0.01–0.01]	0.01 [0.01–0.32]	0.79
72 h (pg/mL)	0.01 [0.01–0.01]	0.25 [0.01–4.56]	**0.025**
LDL-C levels			
Baseline (mg/dL)	106 [76–130]	95 [79–112]	0.37
24 h (mg/dL)	78 [56–114]	92 [70–103]	0.36
72 h (mg/dL)	49 [35–70]	72 [60–92]	**0.002**
Absolute reduction at 72 h (mg/dL)	−55 [38–68]	−18 [2–33]	**<0.001**
Relative reduction at 24 h	−22% [16–30]	−1% [−3.8–12]	**<0.001**
Relative reduction at 72 h	−48% [42–61]	−18% [2.3–30]	**<0.001**
Prevalence of patients with LDL-C <55 mg/dL at 72 h	15 (50.0)	3 (10.0)	**<0.001**
ApoB levels			
Baseline (mg/dL)	81 [62–107]	78 [68–96]	0.96
72 h (mg/dL)	51 [35–70]	68 [60–83]	**<0.001**
Relative reduction at 72 h (%)	−34% [29–50]	−11% [−3–25]	**<0.001**

Data are expressed as median [interquartile range] for continuous variables (non-normal distribution) or as absolute numbers (percentages) for categorical variables. ApoB = Apolipoprotein B; LDL-C = Low-Density Lipoprotein Cholesterol; TNF-α = Tumor Necrosis Factor-alpha. Statistically significant *p* values (<0.05) appear in bold.

**Table 3:** Secondary outcomes: Lp(a), hs-CRP and E-selectin levels.

	Evolocumab Arm (n = 30)	Control Arm (n = 30)	*p* Value
Lp(a) levels			
Baseline (mg/dL)	16 [10–30]	20 [11.5–57]	0.15
72 h (mg/dL)	12 [10–33]	28 [13.1–70]	**0.032**
Relative change at 72 h	0% [−9.2–0]	2.61% [0–23.02]	**0.003**
Hs-CRP levels			
Baseline (mg/dL)	0.38 [0.15–0.96]	0.36 [0.15–1.08]	0.80
24 h (mg/dL)	0.95 [0.32–2.18]	0.83 [0.36–2.73]	0.70
72 h (mg/dL)	1.33 [0.69–2.87]	1.75 [0.43–2.94]	0.74
Relative increase at 72 h	+181.1% [+20.7/+575.7]	+103.3% [+16.1/+349.0]	0.29
E-selectin levels			
Baseline (ng/mL)	24.48 [17.84–33.22]	24.88 [18.05–33.07]	0.81
24 h (ng/mL)	24.59 [18.70–30.76]	25.01 [19.24–38.14]	0.53
72 h (ng/mL)	26.06 [16.00–36.10]	24.46 [18.26–36.76]	0.72
Relative change at 72 h	+2.7% [−15.93/+17.75]	−4.1% [−12.0/+9.1]	0.94

Data are expressed as median [interquartile range] (continuous variables with non-normal distribution). Hs-CRP = High-sensitivity C-Reactive Protein; Lp(a) = Lipoprotein(a). Statistically significant *p* values (<0.05) appear in bold.

**Table 4:** Outcomes at 30 days.

	Evolocumab Arm (n = 30)	Control Arm (n = 30)	*p* Value
MACE	-	-	
All-cause death	1 (3.3)	0 (0)	0.31
LDL-C levels (mg/dL)	12 [8–18]	41 [33–50]	**<0.001**
Patients achieving the LDL-C goal (<55 mg/dL)	27 (90)	23 (77)	0.17

Data are expressed as median [interquartile range] for continuous variables (non-normal distribution) or as absolute numbers (percentages) for categorical variables. LDL-C = Low-Density Lipoprotein Cholesterol; MACE = Major adverse cardiovascular events (cardiovascular death, myocardial infarction, stroke, unplanned coronary revascularization). Statistically significant *p* values (<0.05) appear in bold.

## Data Availability

The data that support the findings of this study are available from the corresponding author upon reasonable request.

## Supplementary Materials

The following supporting information can be downloaded at: https://www.mdpi.com/article/10.3390/jcm15134873/s1, Supplementary Figure S1. Study flow diagram illustrating patient enrollment, allocation to treatment groups, follow-up, and inclusion in the final analysis; Supplementary Table S1. Friedman Test within-group changes across 3 time points (Baseline → 24 h → 72 h); Supplementary Table S2. Wilcoxon Signed-Rank Test: paired within-group changes across 2 time points; Supplementary Table S3. Subgroup analyses.
