# Supplementary material for: Suppression of Early TNF-Alpha Increase by a Single Evolocumab Dose in Patients with Acute Myocardial Infarction Undergoing Percutaneous Coronary Intervention
Source: J Clin Med. 2026 Jun 23;15(13):4873. doi: 10.3390/jcm15134873 (PMC13362465; doi:10.3390/jcm15134873)
Supplement: Supplementary file 1 [file jcm-15-04873-s001.zip › Supplementary Table S1.pdf]

Supplementary Table S1. Friedman Test within-group changes across 3 time points (Baseline → 24h → 72h).

Parameter	Arm	Timepoints	χ^2 Statistic	P value
**TNF-α**	Evolocumab arm (n=30)	Baseline / 24h / 72h	1.22	0.544
**TNF-α**	Control arm (n=30)	Baseline / 24h / 72h	13.56	**0.001**
**hs-CRP**	Evolocumab arm (n=30)	Baseline / 24h / 72h	14.62	**<0.001**
**hs-CRP**	Control arm (n=30)	Baseline / 24h / 72h	14.07	**<0.001**
**E-selectin**	Evolocumab arm (n=30)	Baseline / 24h / 72h	0.80	0.670
**E-selectin**	Control arm (n=30)	Baseline / 24h / 72h	2.48	0.289

hs-CRP= High sensitivity C-reactive protein; TNF-α= Tumor Necrosis Factor-alpha.
