# Supplementary material for: Suppression of Early TNF-Alpha Increase by a Single Evolocumab Dose in Patients with Acute Myocardial Infarction Undergoing Percutaneous Coronary Intervention
Source: J Clin Med. 2026 Jun 23;15(13):4873. doi: 10.3390/jcm15134873 (PMC13362465; doi:10.3390/jcm15134873)
Supplement: Supplementary file 1 [file jcm-15-04873-s001.zip › Supplementary Table S2.pdf]

Supplementary Table S2. Wilcoxon Signed-Rank Test: paired within-group changes across 2 time points.

Parameter	Arm	Timepoints	Timepoint 1	Timepoint 2	Paired diff	P value
**TNF-α**	Evolocumab arm (n=30)	Baseline vs 24h	0.01 [0.01–0.01]	0.01 [0.01–0.01]	0.00 [0.00–0.00]	0.169
**TNF-α**	Evolocumab arm (n=30)	Baseline vs 72h	0.01 [0.01–0.01]	0.01 [0.01–0.01]	0.00 [0.00–0.00]	0.959
**TNF-α**	Evolocumab arm (n=30)	24h vs 72h	0.01 [0.01–0.01]	0.01 [0.01–0.01]	0.00 [0.00–0.00]	0.638
**TNF-α**	Control arm (n=30)	Baseline vs 24h	0.01 [0.01–0.01]	0.01 [0.01–0.32]	0.00 [0.00–0.00]	0.093
**TNF-α**	Control arm (n=30)	Baseline vs 72h	0.01 [0.01–0.01]	0.25 [0.01–4.56]	0.00 [−2.29–0.00]	0.053
**TNF-α**	Control arm (n=30)	24h vs 72h	0.01 [0.01–0.32]	0.25 [0.01–4.56]	0.00 [−1.29–0.00]	0.074
**LDL-C**	Evolocumab arm (n=30)	Baseline vs 24h	106 [76–130]	78 [58–110]	20 [13–31]	**<0.001**
**LDL-C**	Evolocumab arm (n=30)	Baseline vs 72h	110 [78–132]	49 [36–70]	55 [38–67]	**<0.001**
**LDL-C**	Evolocumab arm (n=30)	Baseline vs 1 month	109 [79–130]	12 [8–18]	90 [70–120]	**<0.001**
**LDL-C**	Control arm (n=30)	Baseline vs 24h	95 [79–110]	92 [70–103]	1 [−5–10]	0.358
**LDL-C**	Control arm (n=30)	Baseline vs 72h	95 [79–112]	72 [60–91]	18 [2–32]	**<0.001**
**LDL-C**	Control arm (n=30)	Baseline vs 1 month	95 [80–112]	41 [33–50]	63 [39–70]	**<0.001**

**ApoB**	Evolocumab arm (n=30)	Baseline vs 72h	84 [63–108]	51 [35–68]	30 [23–37]	**<0.001**
**ApoB**	Control arm (n=30)	Baseline vs 72h	78 [70–96]	68 [60–82]	9 [−2–22]	**0.003**
**Lp(a)**	Evolocumab arm (n=30)	Baseline vs 72h	15.9 [10.0–30.0]	12.2 [10.0–31.5]	0.00 [0.00–4.65]	0.140
**Lp(a)**	Control arm (n=30)	Baseline vs 72h	19.7 [11.5–56.9]	28.3 [13.1–70.0]	−0.30 [−7.10–0.00]	**0.006**
**hs-CRP**	Evolocumab arm (n=30)	Baseline vs 24h	0.38 [0.15–0.96]	0.95 [0.34–2.18]	−0.28 [−1.00–−0.08]	**<0.001**
**hs-CRP**	Evolocumab arm (n=30)	Baseline vs 72h	0.41 [0.15–1.12]	1.33 [0.69–2.71]	−0.46 [−1.82–−0.10]	**0.001**
**hs-CRP**	Control arm (n=30)	Baseline vs 24h	0.36 [0.16–1.06]	0.83 [0.39–2.69]	−0.23 [−1.35–−0.02]	**0.002**
**hs-CRP**	Control arm (n=30)	Baseline vs 72h	0.36 [0.16–1.06]	1.75 [0.45–2.87]	−0.24 [−1.69–0.03]	**0.033**

Data are expressed as median [interquartile range] for continuous variables (non-normal distribution).

hs-CRP= High sensitivity C-reactive protein; LDL-C= Low-Density Lipoprotein Cholesterol; TNF-α= Tumor Necrosis Factor-alpha
