# Supplementary material for: Suppression of Early TNF-Alpha Increase by a Single Evolocumab Dose in Patients with Acute Myocardial Infarction Undergoing Percutaneous Coronary Intervention
Source: J Clin Med. 2026 Jun 23;15(13):4873. doi: 10.3390/jcm15134873 (PMC13362465; doi:10.3390/jcm15134873)
Supplement: Supplementary file 1 [file jcm-15-04873-s001.zip › Supplementary Table S3.pdf]

Supplementary Table S3. Subgroup analyses.

	**Evolocumab arm** (n=30)	**Control arm** (n=30)
TNF-α levels – NSTEMI patients		
Baseline (pg/mL)	0.01 [0.01 - 0.01]	0.01 [0.01 - 0.01]
72 hours (pg/mL)	0.01 [0.01 - 0.01]	0.25 [0.01 – 4.56]
TNF-α levels – STEMI patients		
Baseline (pg/mL)	0.01 [0.01 - 0.01]	0.01 [0.01 - 0.17]
72 hours (pg/mL)	0.01 [0.01 - 0.01]	0.59 [0.01 – 7.10]
LDL-C relative change at 72 hours vs baseline – Chronic statin therapy yes	- 54% [46 – 63]	- 7% [-8 – 23]
LDL-C relative change at 72 hours vs baseline – Chronic statin therapy no	- 44% [34 – 57]	- 21% [7 – 32]

Data are expressed as median [interquartile range] for continuous variables (non-normal distribution)

LDL-C= Low-Density Lipoprotein Cholesterol; NSTEMI= Non ST-segment Elevation Myocardial Infarction; STEMI= ST-segment Elevation Myocardial Infarction; TNF-α= Tumor Necrosis Factor-alpha
