# Supplementary figures and images for: Suppression of Early TNF-Alpha Increase by a Single Evolocumab Dose in Patients with Acute Myocardial Infarction Undergoing Percutaneous Coronary Intervention
Source: J Clin Med. 2026 Jun 23;15(13):4873. doi: 10.3390/jcm15134873 (PMC13362465; doi:10.3390/jcm15134873)

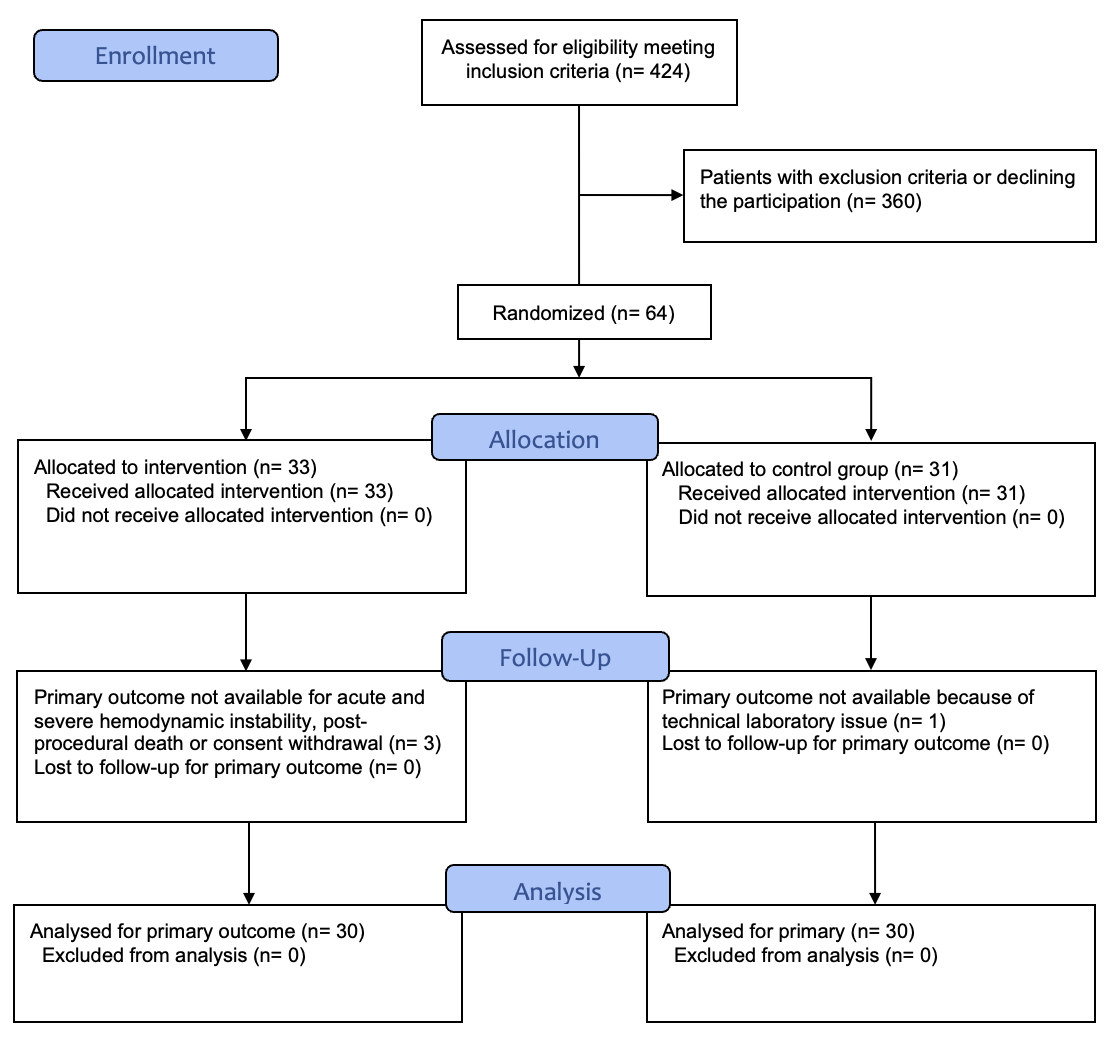

Supplement: Supplementary file 1 [file jcm-15-04873-s001.zip › Supplementary Figure S1.tiff]
